# Dynamics of Urinary Calprotectin after Renal Ischaemia

**DOI:** 10.1371/journal.pone.0146395

**Published:** 2016-01-08

**Authors:** Jan Ebbing, Felix S. Seibert, Nikolaos Pagonas, Frederic Bauer, Kurt Miller, Carsten Kempkensteffen, Karsten Günzel, Alexander Bachmann, Hans H. Seifert, Cyrill A. Rentsch, Peter Ardelt, Christian Wetterauer, Patrizia Amico, Nina Babel, Timm H. Westhoff

**Affiliations:** 1 University Hospital Basel, Department of Urology, Basel, Switzerland; 2 Charité - University Hospital, Campus Benjamin Franklin, Department of Urology, Berlin, Germany; 3 University Hospital Marien Hospital Herne, Medical Department I, Ruhr University of Bochum, Bochum, Germany; 4 University Hospital Basel, Department of Nephrology, Basel, Switzerland; Johannes Kepler University Linz, AUSTRIA

## Abstract

Background: Urinary calprotectin has been identified as a promising biomarker for acute kidney injury. To date, however, the time-dependent changes of this parameter during acute kidney injury remain elusive. The aim of the present work was to define the time-course of urinary calprotectin secretion after ischaemia/reperfusion-induced kidney injury in comparison to neutrophil gelatinase—associated lipocalin, thereby monitoring the extent of tubular damage in nephron sparing surgery for kidney tumours. Methods: The study population consisted of 42 patients. Thirty-two patients underwent either open or endoscopic nephron sparing surgery for kidney tumours. During the surgery, the renal arterial pedicle was clamped with a median ischaemic time of 13 minutes (interquartile range, 4.5–20.3 minutes) in 26 patients. Ten retro-peritoneoscopic living donor nephrectomy patients and 6 nephron sparing surgery patients in whom the renal artery was not clamped served as controls. Urinary calprotectin and neutrophil gelatinase—associated lipocalin concentrations were repeatedly measured by enzyme-linked immunosorbent assay and assessed according to renal function parameters. Results: Urinary concentrations of calprotectin and neutrophil gelatinase—associated lipocalin increased significantly after ischaemia/reperfusion injury, whereas concentrations remained unchanged after nephron sparing surgery without ischaemia/reperfusion injury and after kidney donation. Calprotectin and neutrophil gelatinase—associated lipocalin levels were significantly increased 2 and 8 hours, respectively, post-ischaemia. Both proteins reached maximal concentrations after 48 hours, followed by a subsequent persistent decrease. Maximal neutrophil gelatinase—associated lipocalin and calprotectin concentrations were 9-fold and 69-fold higher than their respective baseline values. The glomerular filtration rate was only transiently impaired at the first post-operative day after ischaemia/reperfusion injury (p = 0.049). Conclusion: Calprotectin and neutrophil gelatinase—associated lipocalin can be used to monitor clinical and sub-clinical tubular damage after nephron sparing surgery for kidney tumours. Urinary calprotectin concentrations start rising within 2 hours after ischaemia/reperfusion-induced kidney injury.

## Introduction

Calprotectin in the urine has recently been identified as a promising biomarker for acute kidney injury (AKI) [1,2]. It can differentiate between intrinsic and prerenal causes of AKI. Calprotectin is a calcium-binding complex comprising 2 proteins of the so-called S100 group (S100A8/S100A9). Calprotectin is a mediator protein of the innate immune system calprotectin, and it is released by monocytes and neutrophils as a danger-associated molecular pattern protein [3]. Additionally, renal collecting duct epithelial cells produce S100A8 and S100A9 in response to renal injury [4]. Whereas calprotectin levels in prerenal disease are comparable to healthy controls, intrinsic AKI leads to highly increased calprotectin concentrations. In a study population of 188 subjects, calprotectin achieved a high diagnostic performance in the differentiation of intrinsic and prerenal AKI. Urinary calprotectin concentrations were 50-fold higher in intrinsic AKI than in prerenal AKI. In that study population, calprotectin achieved a higher diagnostic performance than neutrophil gelatinase—associated lipocalin (NGAL) in the differentiation between prerenal and intrinsic AKI [1]. Calprotectin is not only an AKI marker; it can also mediate AKI. In a S100A9-knockout mouse model, it was recently demonstrated that calprotectin played a crucial role in renal repair after ischaemia/reperfusion-induced kidney injury: S100A8/S100A9 inhibited M2-polarization of macrophages, thereby preventing the induction of renal fibrosis and damage after AKI [5].

The prognosis of AKI crucially depends on the early and correct identification of the underlying condition. The earlier the treatment, the better the chance of ameliorating the renal function impairment [6]. Whereas post-renal AKI is easily detectable by ultrasound, the differentiation between prerenal and intrinsic disease can be challenging. So far, there has been no reliable biomarker capable of differentiating between these 2 conditions. Therefore, the identification of calprotectin as a diagnostic marker may be of practical clinical interest. Adequate clinical use of a diagnostic parameter, however, necessitates detailed knowledge on the time-dependent changes of this parameter after renal injury. For example, it takes up to 2 days for the levels of the most frequently used AKI biomarker, creatinine, to begin to rise. Therefore, the identification of NGAL as an early marker of AKI evoked considerable interest. Elevated urinary NGAL concentrations were reached within hours following ischaemic renal injury [7–9]. Hence, NGAL is regarded as a kind of “troponin of the kidney” [10]. However, the time-course of calprotectin secretion in the urine after renal injury remains elusive.

The present study makes use of nephron sparing surgery (NSS) for kidney tumours as a model for ischaemia/reperfusion-induced tubular damage. In the majority of cases, NSS requires clamping of the renal artery for several minutes, thereby providing the opportunity to examine the effects of ischaemia and reperfusion in an *in vivo* setting. Subjects undergoing NSS without clamping of the renal artery served as controls. Thus, this approach allowed the first characterisation of the time-course of urinary calprotectin secretion in tubular injury in comparison to NGAL.

## Methods

### Study population

This study was approved by the local ethics committee of the Charité –University Hospital Berlin and by the ethics committee of Northwest und Central Switzerland. All patients provided written informed consent. Patients were referred to the Department of Urology at the Charité –University Hospital Berlin or to the Department of Urology at the University Hospital Basel for surgical treatment of renal tumours by NSS or living donor nephrectomy. The study population included 42 patients, divided into 3 groups: 1 group of patients with renal ischaemia/reperfusion injury and 2 control groups.

Group 1 (iatrogenic renal ischaemia/reperfusion injury, n = 26): patients undergoing NSS for renal tumours. The inclusion criterion was a resection of a solid renal mass necessitating a temporary interruption of the renal arterial blood flow to control bleeding.

Group 2 (control group, no renal ischaemia, n = 6): patients undergoing NSS for renal tumours without the need for renal artery clamping.

Group 3 (control group, no renal ischaemia, no surgery-related mechanical damage to the remaining kidney, n = 10): patients undergoing nephrectomy as a living kidney donor.

For all 3 groups, exclusion criteria comprised pre-existing impairments of renal function with an estimated glomerular filtration rate (eGFR) < 50 ml/min according to the Chronic Kidney Disease Epidemiology Collaboration (CKD-EPI) formula [11], urinary tract infections, and bladder cancers. AKI was defined according to the Acute Kidney Injury Network (AKIN) criteria as an increase in plasma creatinine levels ≥ 50% or ≥ 0.3 mg/dl [12].

### Surgical technique

NSS (Groups 1 and 2) was performed by open surgery, laparoscopy, retro-peritoneoscopy, or robot-assisted laparoscopic surgery. Living donor nephrectomy (Group 3) was conducted retro-peritoneoscopically. In Group 1, surgery-related renal ischaemia was performed by clamping the arterial renal pedicle with Satinsky vascular clamps in open surgery, or with Bulldog vascular clamps in endoscopic surgery. Indications for arterial renal pedicle clamping were determined by the surgeon. The length of time of the blood flow interruption was documented (clamp time). Nephro-protective methods, such as induction of surface hypothermia of the kidney with ice slush or intravenous mannitol injections, were not applied.

### Sample collection

At the beginning of the surgical procedure, a urinary indwelling catheter was placed and a primary urine sample was obtained for the assessment of baseline urinary calprotectin and NGAL concentrations. During the intra- and post-operative courses, urine samples for the sequential assessment of urinary calprotectin and NGAL were obtained after surgical kidney exposure directly before clamping of the renal artery (Group 1), directly before tumour resection (Group 2), or directly before vessel and ureter ligation (Group 3). Urine samples were also collected in all 3 groups at the end of the operation (approximately 2 h post-ischaemia). Thereafter, samples were obtained 6 h post-operatively (approximately 8 h post-ischaemia) and once daily for the first 5 post-operative days. Plasma creatinine levels were measured prior to the procedure, 6 h post-operatively (approximately 8 h post-ischaemia) and once daily for the first 5 postoperative days in all groups. In a small number of patients, we could not analyse samples at every time point. In the overall study population, 13.5% (interquartile range, 5.3–18.5%) of urinary calprotectin/NGAL samples are missing for each measurement point.

### Measurement of urinary calprotectin and NGAL concentrations

Collected samples were stored at -20°C, without centrifugation, until calprotectin and NGAL concentrations were assessed. Urine concentrations of calprotectin were quantified using the PhiCal^®^ Calprotectin enzyme-linked immunosorbent assay (ELISA) kit (Immundiagnostik AG, Bensheim, Germany) according to the manufacturer’s protocol, as previously published [1]. Concentrations of NGAL were assessed using the NGAL Rapid ELISA Kit (Bioporto, Gentofte, Denmark), as previously described [1]. This assay has been clinically validated [13]. We did not assess urinary creatinine levels, as we have previously shown that the diagnostic performance of urinary calprotectin concentrations was not improved when adjusted for urinary creatinine [1,2].

### Statistical analysis

Continuous data were presented as the median and interquartile range (IQR). Data on an ordinal or continuous level were analysed with the non-parametric Mann-Whitney-U test when there was a comparison between 2 groups (e.g. Group 1 versus Group 2), and with the Kruskal-Wallis test when there were > 2 groups (e.g. Group 1 versus Group 2 versus Group 3). A comparison of categorical parameters was performed using Fisher´s exact test. A non-parametric analysis of variance (Kruskal-Wallis test, corrected for multiple comparisons with Dunn´s test as a post hoc pairwise multiple comparison test) was used to test for significant changes from baseline in the levels of calprotectin, NGAL, and plasma creatinine, and eGFR over the course of the observation period. In accordance with the results of a preceding Shapiro-Wilk normality test and Q-Q plot, a non-normality Spearman’s correlation test was applied to determine the association between urinary calprotectin or NGAL and ischaemic time or eGFR in Group 1. A linear regression test was performed to define independent prognostic factors for the maximum increase of urinary calprotectin and NGAL in Group 1. A p-value < 0.05 was regarded as statistically significant. All statistical analyses were performed using SPSS Statistics 19 (SPSS Inc., Chicago, Illinois, USA) or Graphpad Prism 6 (GraphPad Software, Inc., La Jolla, California, USA).

## Results

Thirty-two patients who underwent NSS (Groups 1 and 2) and 10 patients who underwent a living donor nephrectomy (Group 3) were included in this study. Epidemiological data, concomitant diseases, tumour characteristics, surgical data, renal parameters, and baseline urinary calprotectin and NGAL concentrations for these patients are presented in Table 1. Clamping of the renal artery during the surgical procedure was necessary in 26 patients (Group 1). The median renal pedicle clamp time (ischaemic time) was 13.0 min (IQR, 4.5–20.3 min).

**Table 1:** Epidemiological data, concomitant diseases, tumour characteristics, surgical data, renal parameters, and baseline urinary calprotectin/NGAL concentrations in the study population.

	Group 1	Group 2	Group 3	p-value
	NSS with ischaemia	NSS without ischaemia	Living donor nephrectomy	
	(n = 26)	(n = 6)	(n = 10)	
**Epidemiology**				
Male/Female	21 (80.8%)/5 (19.2%)	2 (33.3%)/4 (66.7%)	4 (40%)/6 (60%)	0.02
Age (years)	64 (54–75.25)	63 (43.25–69)	53 (46.25–61)	0.08
Body mass index (kg/m^2^)	26.6 (24.5–31.5)	24.6 (21.75–25.83)	24 (20–28)	0.07
**Concomitant diseases**				
Diabetes mellitus	5 (19.2%)	0 (0%)	0 (0%)	0.24
Hypertension	16 (61.5%)	3 (50%)	2 (20%)	0.08
Pre-existing CKD	4 (15.4%)	1 (16.7%)	0 (0%)	0.40
Coronary heart disease	3 (11.5%)	2 (33.3%)	0 (0%)	0.16
Status after nephrectomy	2 (7.7%)	1 (16.7%)	0 (0%)	0.58
Status after NSS	2 (7.7%)	1 (16.7%)	0 (0%)	0.58
**Medication on admission**				
ACE-I/ARB	13 (50%)	4 (66.7%)	1 (10%)	0.04
Diuretics	6 (23.1%)	1 (16.7%)	0 (0%)	0.22
**Side**				
Right/Left kidney	10 (38.5%)/16 (61.5%)	3 (50%)/3 (50%)	1 (10%)/9 (90%)	0.24
**Tumour characteristics**				
Type of tumour				0.19
RCC	23 (88.5%)	4 (66.7%)	-	
Oncocytoma	2 (7.7%)	1 (16.7%)	-	
Angiomyolipoma	0 (0%)	1 (16.7%)	-	
Other	1 (3.8%)	0 (0%)	-	
RCC histology				0.11
clear cell	17 (65.4%)	1 (16.7%)	-	
papillary	2 (7.7%)	2 (33.3%)	-	
chromophobic	4 (15.4%)	1 (16.7%)	-	
Staging of RCC				0.89
pT1a	17 (65.4%)	3 (50%)	-	
pT1b	5 (19.2%)	0 (0%)	-	
pT2a	0 (0%)	1 (16.7%)	-	
pT2b	1 (3.8%)	0 (0%)	-	
Tumour diameter (mm)	35 (22.7–41.25)	24.5 (12–79.25)	-	0.54
**Surgical data**				
Operation time (min)	212.5 (177.25–260)	154 (96.25–180.75)	183 (163.75–207.5)	0.003
Renal artery clamping (ischaemia)	26 (100%)	0 (0%)	-	
Ischaemic time (min)	13 (4.5–20.25)	-	-	
Surgical approach				0.75 ^a^
Open surgery	15 (57.7%)	3 (50%)	0 (0%)	
Endoscopic surgery	11 (42.3%)	3 (50%)	10 (100%)	
Laparoscopy (lap.)	6 (23.1%)	1 (16.7%)	0 (0%)	
Robot-assisted lap.	4 (15.4%)	2 (33.3%)	0 (0%)	
Retro-peritoneoscopy	1 (3.8%)	0 (0%)	10 (100%)	
**Renal data**				
eGFR on admission ^b^ (ml/min/1.73 m^2^)	84.6 (66.3–97.8)	71.4 (52.6–91.6)	96.4 (89.25–109.5)	0.37
Plasma creatinine on admission (mg/dl)	0.91 (0.83–1.01)	0.85 (0.77–1.49)	0.76 (0.65–0.84)	0.73
**Urinary biomarkers**				
Urinary calprotectin on admission (ng/ml)	41.72 (11.07–148.82)	78.91 (54.65–268.79)	36.35 (3.83–306.55)	0.17
Urinary NGAL on admission (ng/ml)	7.04 (2.85–13.84)	12.10 (4.84–18.65)	5.38 (3.20–9.3)	0.56

NSS, nephron sparing surgery of kidney tumours; CKD, chronic kidney disease; ACE-I/ARB, angiotensin-converting enzyme inhibitor/angiotensin receptor blocker; RCC, renal cell cancer; eGFR, estimated glomerular filtration rate; NGAL, neutrophil gelatinase-associated lipocalin.

Continuous data are presented as median and interquartile range.

^a^ applies only to NSS groups.

^b^ calculated according to CKD-EPI (Chronic Kidney Disease Epidemiology Collaboration) formula.

**Fig 1:**
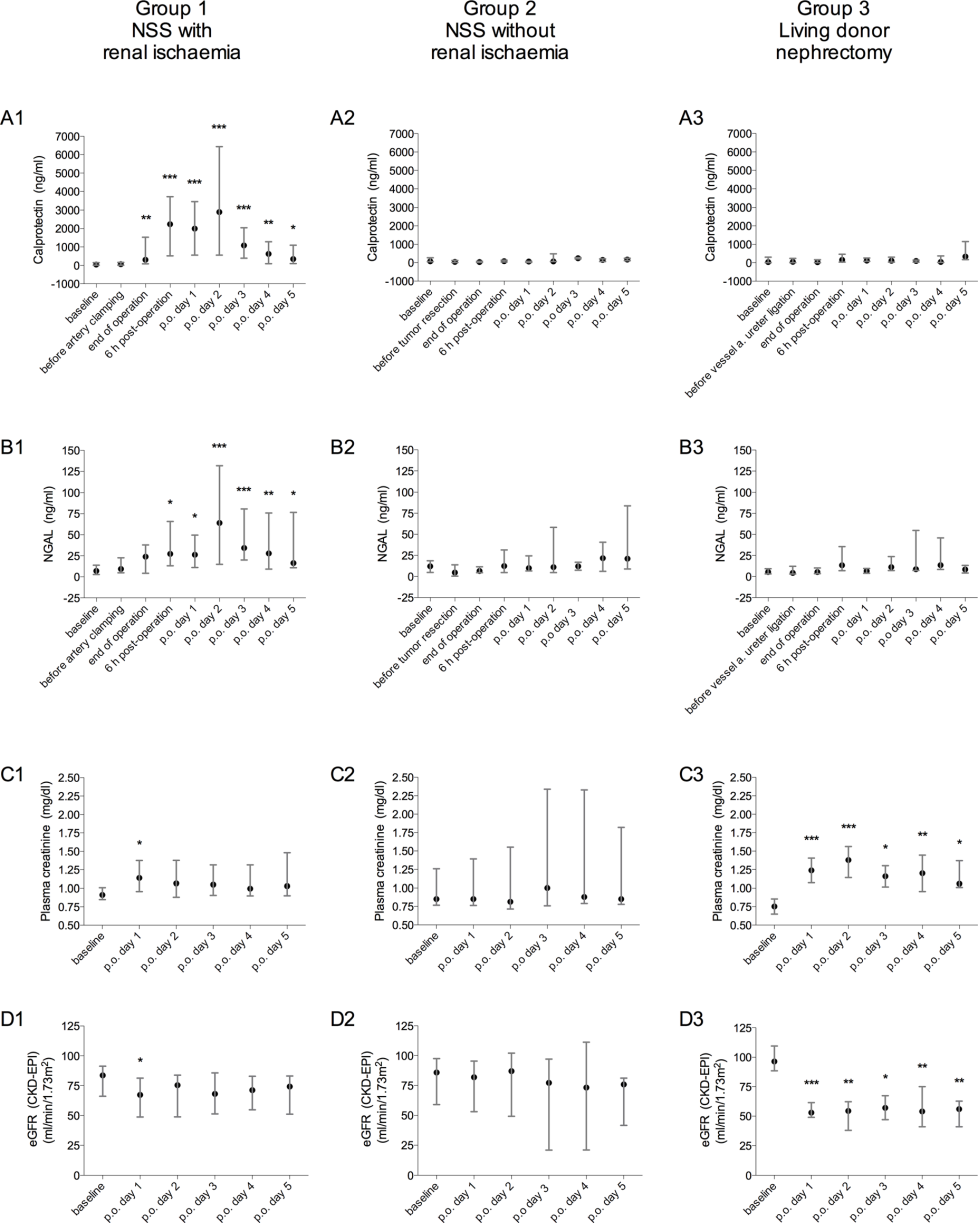
Concentrations of (A) urinary calprotectin, (B) urinary neutrophil gelatinase—associated lipocalin (NGAL), (C) plasma creatinine, and (D) the estimated glomerular filtration rate (eGFR) in patients undergoing nephron sparing surgery (NSS) for kidney tumours with renal pedicle clamping (renal ischaemia, Group 1) or without renal ischaemia (Group 2), and in patients undergoing radical nephrectomy for living kidney donation (Group 3). Data are presented as median and interquartile range. Asterisks indicate significant changes from baseline in a non-parametric analysis of variance (Kruskal-Wallis test, corrected for multiple comparisons by Dunn’s test as a post hoc pairwise multiple comparison). p.o., post-operative. * p < 0.05, ** p < 0.01, *** p < 0.001.

As presented in Fig 1/D1, the post-operative eGFR in Group 1 was slightly, but significantly, decreased from 83.6 ml/min (IQR, 66.1–91.4 ml/min) to 67.2 ml/min (IQR, 48.7–81.4 ml/min, Δ 16.4 ml/min, p = 0.049) after 24 h (post-operative day 1). Although eGFR was also decreased 6 h post-operatively, this was not significant (Δ 7.0 ml/min, p = 0.98, data not shown). Renal function was recovered within 48 h (postoperative day 2) with eGFR of 75.4 ml/min (IQR, 48.8–83.8 ml/min). At discharge from the hospital, eGFR was 9.4 ml/min lower than the baseline rate (p = 0.29). According to the AKIN criteria, 9/26 Group 1 patients, 1/6 Group 2 patients, and 9/10 Group 3 patients developed AKI.

There was a significant increase in both urinary calprotectin (p < 0.001) and NGAL (p < 0.001) levels in response to renal ischaemia (Fig 1/A1 and 1/B1). Calprotectin was the first to show a significant increase: approximately 2 h post-ischaemia, at the end of the operation (Δ 263.1 ng/ml, p = 0.009). NGAL levels were significantly increased 6 h post-operation (approximately 8 h post-ischaemia, Δ 20.3 ng/ml, p = 0.01). Maximal NGAL and calprotectin concentrations were reached at 48 h (post-operative day 2). NGAL increased from 7.0 ng/ml (IQR, 2.9–13.8 ng/ml) to 64.0 ng/ml (IQR, 14.9–131.7 ng/ml). Urinary calprotectin levels increased from 41.7 ng/ml (IQR, 11.1–148.8 ng/ml) to 2894.0 ng/ml (IQR, 558.7–6436.0 ng/ml) within 48 h, after which the levels began to decrease. The maximal NGAL and calprotectin concentrations were 9-fold and 69-fold higher than baseline values. At discharge, NGAL concentrations were still significantly higher than baseline levels (7.0 ng/ml [IQR, 2.9–13.9 ng/ml] vs. 16.4 ng/ml [IQR, 10.9–76.7 ng/ml], p = 0.01). Urinary calprotectin concentrations returned to 345.0 ng/ml (IQR, 96.82–1096.0 ng/ml) by post-operative day 5 (120 h). Calprotectin levels at discharge were still significantly different from the pre-ischaemia levels (Δ 303.3 ng/ml, p = 0.02).

In Groups 2 and 3, there were no significant changes in urinary calprotectin and NGAL concentrations. In Group 2, renal function remained stable post-operatively with no significant changes in plasma creatinine levels or eGFR (Fig 1/C2 and 1/D2). In Group 3, eGFR were significantly decreased from 96.4 ml/min (IQR, 88.5–109.5 ml/min) at baseline to 53.0 ml/min (IQR, 49.0–61.5 ml/min) at post-operative day 1, and remained low until post-operative day 5 (Fig 1/D3); this decrease was a result of the kidney donation.

**Fig 2:**
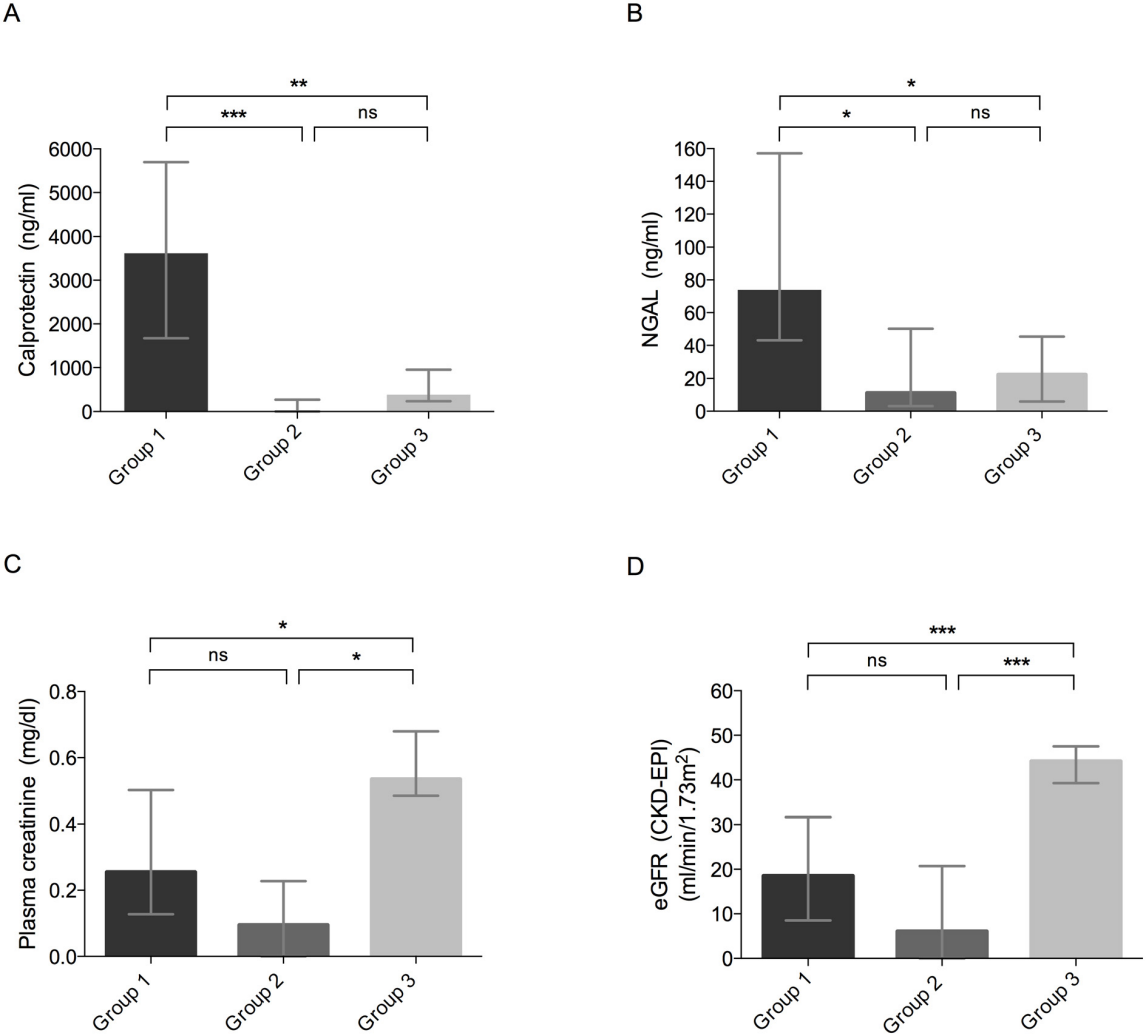
Maximal change from baseline of (A) urinary calprotectin, (B) urinary NGAL, (C) plasma creatinine, and (D) eGFR in patients undergoing NSS with renal renal ischaemia (Group 1) and NSS without renal ischaemia (Group 2), and in patients undergoing nephrectomy for living kidney donation (Group 3). Data are presented as median and interquartile range. Asterisks indicate significant differences in a non-parametric analysis of variance (Kruskal-Wallis test, corrected for multiple comparisons by Dunn’s test as a post hoc pairwise multiple comparison test). NSS, nephron sparing surgery for kidney tumours; eGFR, estimated glomerular filtration rate. ^ns^ not significant, * p < 0.05, ** p < 0.01, *** p < 0.001.

Fig 2 presents the maximal changes in calprotectin, NGAL, and plasma creatinine concentrations, and eGFR within the 3 groups. Maximal concentrations of calprotectin and NGAL were significantly higher in Group 1 than in Groups 2 and 3 (calprotectin 3665.0 ng/ml [IQR, 1671.0–5698.0 ng/ml] versus 0.0 ng/ml [IQR, 0.0–273.0 ng/ml] versus 433.5 ng/ml [IQR, 239.4–950.6 ng/ml], p < 0.001, Fig 2A; NGAL 75.1 ng/ml [IQR, 43.1–157.2 ng/ml] versus 11.1 ng/ml [IQR, 3.1–50.2 ng/ml] versus 22.3 ng/ml [IQR, 6.0–45.5 ng/ml], p = 0.0013, Fig 2B). Changes in kidney function, as assessed by the maximum increase in plasma creatinine levels and the maximum decrease in eGFR, were not significantly different in Groups 1 and 2 (p = 0.16 and p = 0.23, respectively). There was, however, a significant difference between Groups 1 and 3 (p = 0.01 and p < 0.001, respectively) (Fig 2C and 2D).

Table 2 presents the univariate linear regression analysis for prognostic factors of the maximum calprotectin and NGAL increases in Group 1.

**Table 2:** Univariate linear regression analysis for prognostic factors of the maximum urinary calprotectin increase (*x*—*italics top line*) and the maximum urinary NGAL increase (y—bottom line) in Group 1 patients (NSS with renal ischaemia).

Covariate		Regression Co-efficient B	Correlation Co-efficient R	Correlation Co-efficient R^2^	95% CI of B	p-value
Ischaemia time (min) ^a^	*x*	*103*.*61*	*0*.*16*	*0*.*03*	*- 219*.*96–427*.*19*	*0*.*51*
	y	1.71	0.09	0.01	- 7.88–11.31	0.71
Ischaemia duration < or > 13 min	*x*	*2026*.*62*	*0*.*22*	*0*.*05*	*- 2471*.*17–6524*.*40*	*0*.*36*
	y	84.45	0.31	0.10	- 44.43–213.34	*0*.*19*
Ischaemia duration < or >20 min	*x*	*- 1224*.*16*	*0*.*12*	*0*.*02*	*- 6113*.*30–3664*.*98*	*0*.*61*
	y	- 38.53	0.13	0.02	- 182.13–105.07	0.58
Operation time (min) ^a^	*x*	*13*.*01*	*0*.*18*	*0*.*03*	*- 18*.*05–44*.*07*	*0*.*40*
	y	- 0.68	0.19	0.04	- 2.20–0.83	0.40
Renal artery clamping lap./open	*x*	*- 218*.*46*	*0*.*03*	*0*.*001*	*- 3724*.*21–3287*.*29*	*0*.*90*
	y	- 143.64	0.35	0.13	- 303.79–16.51	0.08
Sex m/f	*x*	*- 2696*.*36*	*0*.*18*	*0*.*03*	*- 6184*.*89–2466*.*86*	*0*.*38*
	y	*- 91*.*62*	0.14	0.02	- 284.6–140.29	0.49
Age	*x*	*- 6*.*72*	*0*.*02*	*< 0*.*001*	*- 145*.*44–131*.*99*	*0*.*92*
	y	*- 2*.*23*	0.14	0.02	- 9.94–4.46	0.50
BMI	*x*	*147*.*46*	*0*.*16*	*0*.*03*	*- 247*.*13–542*.*05*	*0*.*45*
	y	*6*.*06*	0.13	*0*.*02*	- 13.38–25.49	0.53
Diabetes mellitus	*x*	*1299*.*21*	*0*.*13*	*0*.*16*	*- 3062*.*78–5661*.*21*	*0*.*55*
	y	*- 0*.*46*	0.001	< 0.001	- 215.07–214.15	1.00
Hypertension	*x*	*2855*.*03*	*0*.*34*	*0*.*11*	*-497*.*03–6207*.*10*	*0*.*09*
	y	- 8.66	0.02	< 0.001	- 182.48–165.15	0.92
Coronary heart disease	*x*	*- 940*.*88*	*0*.*10*	*0*.*01*	*- 4737*.*23–2855*.*47*	*0*.*62*
	y	- 114.59	0.18	0.03	- 374.89–145.72	*0*.*37*
Chronic kidney disease	*x*	*- 1725*.*45*	*0*.*15*	*0*.*02*	*- 6472*.*21–3021*.*31*	*0*.*46*
	y	- 91.69	0.17	0.03	- 322.91–139.53	0.42
Status after nephrectomy	*x*	*- 1037*.*73*	*0*.*07*	*0*.*005*	*- 7525*.*09–5449*.*62*	*0*.*74*
	y	- 50.61	0.07	0.005	- 367.31–266.08	0.74
Status after NSS	*x*	*- 642*.*54*	*0*.*04*	*0*.*002*	*- 7138*.*97–5853*.*89*	*0*.*84*
	y	- 78.49	0.10	*0*.*01*	- 394.18–237.20	0.61
ACE-I and/or ARB	*x*	*131*.*09*	*0*.*16*	*< 0*.*001*	*- 3333*.*67–3595*.*86*	*0*.*94*
	y	- 70.38	0.18	0.03	- 236.93–96.16	0.39
Diuretics	*x*	*10*.*36*	*0*.*001*	*< 0*.*001*	*- 4101*.*90–4122*.*63*	*1*.*00*
	y	- 77.41	0.16	0.03	- 275.49–120.67	0.43
Baseline plasma creatinine (mg/dl)	*x*	*57*.*94*	*0*.*002*	*< 0*.*001*	*- 10215*.*50–10331*.*39*	*0*.*99*
	y	45.82	0.04	0.001	- 455.33–546.97	0.85
Baseline eGFR (CKD-EPI) (ml/min per 1.73 m^2^)	*x*	*11*.*70*	*0*.*05*	*0*.*003*	*- 86*.*30–109*.*69*	*0*.*81*
	y	0.47	0.04	0.002	*- 4*.*32–5*.*25*	0.84
Post-operative AKI ^b^	*x*	*1452*.*79*	*0*.*17*	*0*.*03*	*-2137*.*30–5042*.*88*	*0*.*41*
	y	2.28	0.005	< 0.001	- 175.51–180.06	0.98
Calprotectin at baseline (ng/ml)	*x*	*- 2*.*48*	*0*.*09*	*0*.*01*	*15*.*27–10*.*32*	*0*.*69*
	y	0.24	0.17	0.03	- 0.37–0.86	0.42
Calprotectin at end of operation (ng/ml)	*x*	*0*.*07*	*0*.*02*	*< 0*.*001*	*- 1*.*55–1*.*70*	*0*.*93*
	y	- 0.02	0.11	0.01	- 0.10–0.06	0.60
Calprotectin at p.o. day 1 (ng/ml)	*x*	*- 0*.*15*	*0*.*07*	*0*.*01*	*- 1*.*15–0*.*85*	*0*.*75*
	y	- 0.03	0.29	0.08	- 0.09–0.02	0.21
NGAL at baseline (ng/ml)	*x*	*- 29*.*50*	*0*.*10*	*0*.*01*	*- 159*.*96–100*.*98*	*0*.*64*
	y	- 1.04	0.10	0.009	- 7.78–4.96	0.65
NGAL at end of operation (ng/ml)	*x*	*- 22*.*53*	*0*.*12*	*0*.*02*	*- 104*.*61–59*.*54*	*0*.*58*
	y	- 1.65	0.18	0.03	- 5.63–2.34	0.40
NGAL at p.o. day 1 (ng/ml)	*x*	*- 45*.*81*	*0*.*25*	*0*.*06*	*- 130*.*26–38*.*64*	*0*.*27*
	Y	- 3.50	0.34	0.12	- 8.10–1.11	0.13

m, male; f, female; BMI, body mass index; eGFR, estimated glomerular filtration rate; ACE-I, angiotensin-converting enzyme inhibitor; ARB, angiotensin receptor blocker; lap, laparoscopic; NSS, nephron sparing surgery for kidney tumours; AKI, acute kidney injury; i.o., intra-operative; p.o., post-operative; B, regression co-efficient; R, correlation co-efficient; CI, confidence interval.

^a^ Pearson correlation shows a significant association between ischaemic time and operation time (R = 0.67, p = 0.001).

^b^ AKI was defined as a plasma creatinine increase of > 50% or > 0.3 mg/dl from baseline [12].

The linear regression model did not reveal any other significant prognostic factors for maximal calprotectin or NGAL elevation.

**Fig 3:**
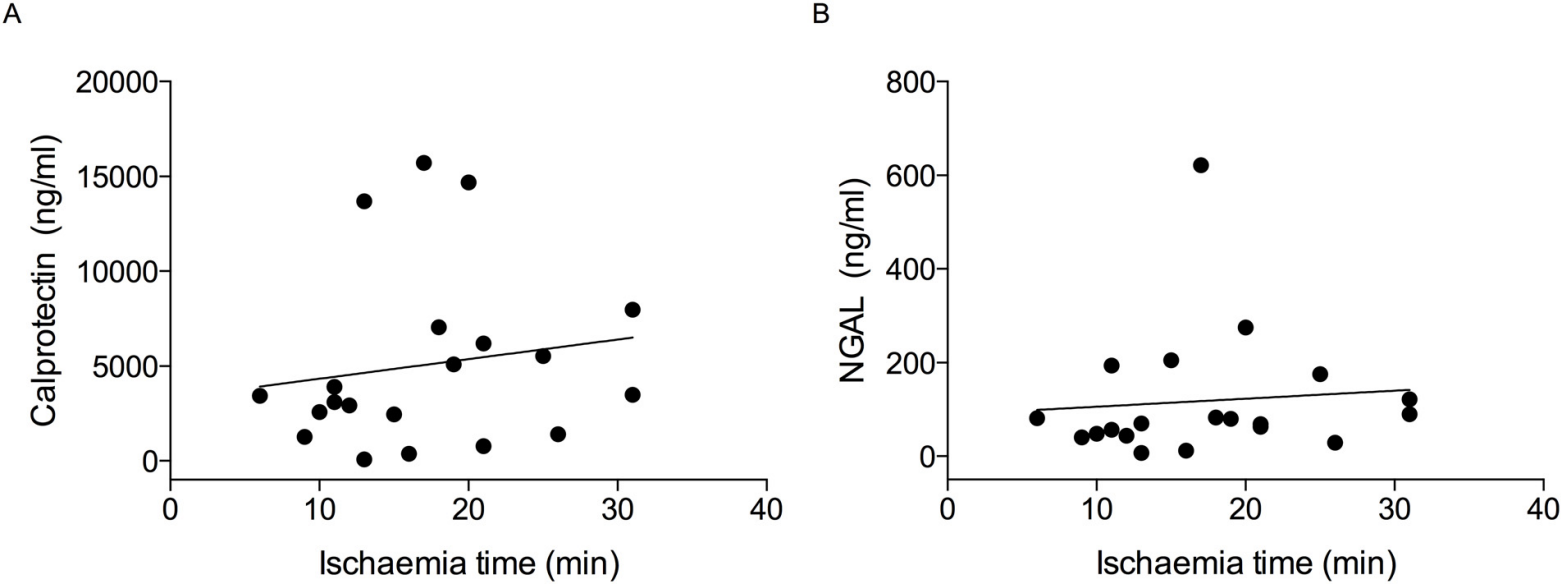
Association between (A) the maximum urinary calprotectin increase and ischaemic time and (B) the maximum urinary NGAL increase and ischaemic time in Group 1 (NSS with renal ischaemia) in a linear regression model, as assessed by Spearman’s correlation test. Regression lines are plotted. Linear regression model: A: R = 0.16, B = 103.61 (95% CI -219.96–427.19), p = 0.51; B: R = 0.09, B = 1.71 (95% CI -7.88–11.31), p = 0.71. Spearman’s correlation: A: R = 0.29 (95% CI -0.19–0.66), p = 0.21; B: R = 0.26 (95% CI -0.22–0.64), p = 0.26. NSS, nephron sparing surgery for kidney tumours; B, regression co-efficient, slope of the regression line; R, correlation co-efficient; CI, confidence interval.

Correspondingly, the extent of the maximum calprotectin and NGAL increases in Group 1 were not associated with ischaemic time, according to Spearman’s correlation (Fig 3).

## Discussion

Calprotectin is a biomarker that differentiates intrinsic from prerenal AKI. To date, however, the time-dependent changes of calprotectin concentrations after intrinsic AKI have remained elusive. A surgical procedure with clamping of the renal artery provides the opportunity to examine the dynamics of renal biomarkers in an *in vivo* ischaemia/reperfusion injury model. The first finding of the present study is that urinary calprotectin concentrations indeed reflect tubular injury. Secondly, urinary calprotectin is more sensitive than plasma creatinine as it detects tubular injury much earlier and with a higher dynamic range than plasma creatinine. Previous studies of calprotectin in AKI comprised heterogeneous patient populations, including those with glomerulonephritis, acute tubular necrosis, pyelonephritis, vasculitis, contrast-induced nephropathy, and interstitial nephritis [1,2]. To the best of our knowledge, our study is the first characterisation of the dynamics of calprotectin secretion in the urine over a defined timeline in a homogeneous population of patients with only ischaemic tubular injury.

Ten years ago, the identification of NGAL initiated the current era of renal biomarker research. In a population of 71 children undergoing a cardio-surgical procedure, it was shown that NGAL could be used to monitor tubular injury. NGAL increased long before tubular injury manifested as a rise in plasma creatinine levels [9]. Hence, NGAL was regarded as a kind of “troponin of the kidney”. It predominantly reflects injury to the distal tubulus and constitutes the best characterised urinary marker of tubular injury so far [10,14]. Therefore, in our current study, we measured NGAL as the “gold standard biomarker” for tubular injury. So far, 4 different study groups have investigated the time-course of changes in urinary NGAL excretion and plasma creatinine levels after renal ischaemia in NSS in humans [7,8,15,16]. In accordance with previous studies [7–9,15,17,18], urinary NGAL significantly increased after renal ischaemia in our population. Hence, the average ischaemic time of 13 min with subsequent reperfusion indeed led to tubular injury in the remaining renal parenchyma. Urinary calprotectin concentrations also reflected this tubular injury. Comparative studies on the secretion of calprotectin in the urine after ischaemic renal injury are not available. Calprotectin concentrations showed a 69-fold increase after the surgical procedure. Interestingly, both calprotectin and NGAL concentrations increased in every single patient in Group 1, thus yielding a sensitivity of 100% for the detection of ischaemia/reperfusion injury. The 2 control groups, who underwent NSS without clamping of the renal artery or with a complete nephrectomy (living donor nephrectomy), revealed that the increase in urinary biomarker concentrations cannot be explained by other peri-procedural factors. Such factors include non-ischaemic surgical kidney trauma, haematuria, leukocyturia, an indwelling catheter, or anaesthesia. Neither calprotectin nor NGAL concentrations changed after the procedure in these control groups. These findings are important since both NGAL and calprotectin concentrations increase in the setting of leukocyturia [1].

Knowledge of the time-dependent dynamics of calprotectin in AKI is essential before it can be potentially implemented in daily clinical practice. The present findings show that concentrations begin to rise shortly after ischaemia. Increases in urinary calprotectin and NGAL concentrations were evident approximately 2 h (end of operation) and 8 h (6 h post-operation), respectively, after ischaemia. The maximum increase in the levels of both proteins was seen after 48 h, with a 69-fold increase over baseline in calprotectin levels and a 9-fold increase over baseline in NGAL levels. One-hundred-and-twenty hours after renal pedicle clamping, both urinary calprotectin and NGAL concentrations were still elevated by 8-fold and 2-fold, respectively. The onset of this increase, thereby, largely precedes any possible changes in plasma creatinine levels, which usually occur > 24 h after kidney injury [9]. The rapid onset of the increase in calprotectin levels is comparable to NGAL. NGAL levels also reached a maximum at day 2 after ischaemia/reperfusion injury. Although the curves seem rather similar, the NGAL increase becomes statistically significant later than the calprotectin increase. With regard to the findings of previous studies, like those of Mishra et al. [9], we must consider the reasons why NGAL levels do not start rising earlier. The average ischaemic time in our study was much shorter (13 min) than the time noted in the analogue studies of Abassi et al. (32 min) [7] and Parekh et al. (23 min) [8]. Consequently, the maximum urinary NGAL concentration 48 h after ischaemia-induced injury in the study from Abassi et al. was approximately twice as high as that in our study. This is a potential explanation for the fact that the increases in NGAL levels became significant earlier in these studies (2–3 h post-ischaemia) than they did in our study. One-hundred-and-twenty hours after renal pedicle clamping, the urinary calprotectin level was still 8-fold higher than the baseline level. Therefore, a “false-negative” calprotectin test result is improbable within 5 days after the onset of intrinsic AKI. In summary, the increase in calprotectin levels in response to ischaemia is rapid, strong, and perpetuates for several days.

In the current study, eGFR is slightly, but significantly, reduced in Group 1 patients at day 1 and resembles baseline values thereafter. This indicates that the ischaemia-induced tubular injury, and perhaps also the resection of the tumour and surrounding functional parenchyma, induced only a slight impairment in renal function. Hence, both NGAL and calprotectin constitute very sensitive markers of tubular injury. Accordingly, calprotectin has been recently shown to sensitively detect ischaemic tubular injury in renal allografts at the time of transplantation [19]. Higher urinary calprotectin concentrations predicted impaired kidney function 4 weeks, 6 months, and 12 months post-transplantation.

With regard to the findings in renal transplant recipients, it appears possible that calprotectin does not only reflect tubular injury in a “black or white” manner, but it may also be able to provide information on the extent of the injury. Due to the minor effects of ischaemia on eGFR in the present study, we could not adequately address this hypothesis in our patient population. Alternatively, we analysed the association between biomarker concentrations and ischaemic time. However, neither maximum calprotectin nor NGAL concentrations were significantly associated with ischaemic time. In the literature, there are inconsistent reports regarding the association between ischaemic time and tubular injury with subsequent loss of renal function [7,8,15,16,18]. The longer the ischaemic time, the more probable it is that an association between this parameter and an NGAL increase could exist. Among all cited studies on urinary NGAL in NSS, only Abassi et al. found a significant association [7,8,16,18].

Are the findings of this study indeed of practical clinical interest? After a decade of research, what is the future perspective for renal biomarkers? In the past 10 years, much effort has been invested in the identification and characterisation of AKI biomarkers. The overwhelming majority of the resulting publications focused on the time-point of the AKI diagnosis in order to improve early diagnosis rates and, hopefully, outcome. To date, several biomarkers, including NGAL and kidney injury molecule, have been identified [10,14,18,20–23]. Indeed, these biomarkers are more sensitive and detect renal damage earlier than conventional markers like plasma creatinine. However, given that we are able to diagnose AKI earlier, what are the clinical benefits of this information? In the setting of surgery or an Intensive Care Unit (ICU), the benefit is “best medical care” with regard to, for example, fluid substitution and blood pressure control. “Best medical care”, however, should be provided independent of any biomarkers. Therefore, the utility of reliable renal biomarkers may be applicable somewhere else. We believe that biomarkers may be of practical clinical help in the differential diagnosis of different forms of AKI. In contrast to the setting of an ICU where the majority of patients have shock- or drug-associated acute tubulus necrosis (ATN), the identification of the cause of AKI in most other clinical situations is difficult. Whereas the diagnosis of post-renal AKI is easily made by ultrasound, the differentiation between prerenal and intrinsic AKI may be difficult. In the absence of a reliable laboratory marker, response to fluid repletion is still regarded as the gold standard for differentiation between these entities. Return of renal function to baseline within 24 to 72 h is considered an indication of prerenal AKI, whereas persistent renal failure indicates intrinsic disease. However, several intrinsic causes, such as crescentic glomerulonephritis, require an immediate biopsy in order to avoid a delay in the initiation of immunosuppressive therapy. In these patients, the 3-day watchful waiting approach before a biopsy procedure is unacceptable. Calprotectin has been proven to be a helpful tool in this context [1,2]. It is largely increased in intrinsic AKI, whereas it is comparable to healthy controls in prerenal disease. Pyuria and urothelial carcinoma can increase calprotectin concentrations independent of AKI and must be taken into account as potential confounders [1,24]. The findings of the present study render further support to the role of calprotectin as a biomarker in AKI, since ATN is a classic example of intrinsic AKI. Calprotectin could be used, for example, as a lateral flow test. A test like this already exists for stool samples. Coming back to the question of whether the present findings can indeed be of clinical significance: yes, they can. Knowledge of the time-dependent dynamics of a biomarker is a crucial pre-requisite for its use in clinical practice. The fact that concentrations are significantly elevated within 2–3 h and remain increased for at least 5 days after the onset of renal injury meets clinical needs.

This study is limited by the duration of the observation period. Since calprotectin concentrations are still increased at day 5 (end of follow-up), it was not possible to determine the length of time it takes for its return to baseline levels. It was not possible to motivate the whole study population to provide further urine samples after discharge from hospital.

The present study shows that ischaemia/reperfusion-induced AKI leads to significantly increased urinary calprotectin concentrations within 2–3 h, reaching a maximum at 48 h with a subsequent decrease thereafter. Levels remain increased for at least 5 days. The detection sensitivity for ischaemia/reperfusion-induced tubular injury was 100% in the present study population. This characterisation of the time-dependent dynamics of this new urinary biomarker is a pre-requisite feature for its potential clinical implementation in the differentiation of prerenal and intrinsic AKI.
